# Vitamin E Isoforms as Modulators of Lung Inflammation

**DOI:** 10.3390/nu5114347

**Published:** 2013-10-31

**Authors:** Hiam Abdala-Valencia, Sergejs Berdnikovs, Joan M. Cook-Mills

**Affiliations:** Allergy-Immunology Division, Feinberg School of Medicine, Northwestern University, McGaw-M304, 240 E. Huron, Chicago, IL 60611, USA; E-Mails: h-abdala-valencia@northwestern.edu (H.A.-V.); s-berdnikovs@northwestern.edu (S.B.)

**Keywords:** tocopherols, lung disease, endothelial

## Abstract

Asthma and allergic diseases are complex conditions caused by a combination of genetic and environmental factors. Clinical studies suggest a number of protective dietary factors for asthma, including vitamin E. However, studies of vitamin E in allergy commonly result in seemingly conflicting outcomes. Recent work indicates that allergic inflammation is inhibited by supplementation with the purified natural vitamin E isoform α-tocopherol but elevated by the isoform γ-tocopherol when administered at physiological tissue concentrations. In this review, we discuss opposing regulatory effects of α-tocopherol and γ-tocopherol on allergic lung inflammation in clinical trials and in animal studies. A better understanding of the differential regulation of inflammation by isoforms of vitamin E provides a basis towards the design of clinical studies and diets that would effectively modulate inflammatory pathways in lung disease.

## 1. Introduction

Asthma is one of the world’s most common chronic diseases, with a conservative estimate of 300 million people being affected by it in 2005 [1]. Typical symptoms include chronic inflammation of the airways, periodic wheezing, breathlessness, paroxysmal cough, and chest tightness, and severity ranges from occasional symptoms to disabling persistent symptoms and/or frequent life-threatening exacerbations. The World Health Organization reported that the prevalence of asthma and allergic diseases is on a dramatic rise worldwide [1]. Among the potential mechanisms that contribute to airway disease is an imbalance of oxidants and antioxidants. In asthma, the recruitment and activation of inflammatory cells in airways results in oxidative stress. Oxidative stress causes further tissue damage in the respiratory system and derangements of the immune system [2]. To balance oxidation, cells evolved antioxidant mechanisms, such as presence of superoxide dismutase, catalase, glutathiones, peroxiredoxins, and vitamins E and C with antioxidant properties. The marked differences in rates of asthma among countries and the documented increase in asthma prevalence over the last 25 years is likely due to changes in our environment or lifestyle because changes in our genetic makeup would take more than several generations to occur [3]. In particular, dietary and environmental factors that diminish the cellular oxidant reducing capacity can increase tissue vulnerability to lung disorders [2].

A number of studies have sought to establish the protective role of the antioxidant vitamin E in asthma. However, these studies have had conflicting outcomes. The focus of this review is to discuss the cellular mechanisms behind differential effects of vitamin E isoforms on airways allergic inflammation. We provide a potential explanation for conflicting outcomes of studies with vitamin E. We discuss the opposing regulatory functions of the vitamin E isoforms α-tocopherol and γ-tocopherol in mice and the mechanisms for anti- and pro-inflammatory functions of these tocopherol isoforms [4,5]. Differences in study outcomes may be based on differences in levels of each of the vitamin E isoforms present in the study supplements, vehicles and diets [4,5,6,7,8,9,10].

## 2. Versatile Nature of Vitamin E

**Figure 1:**
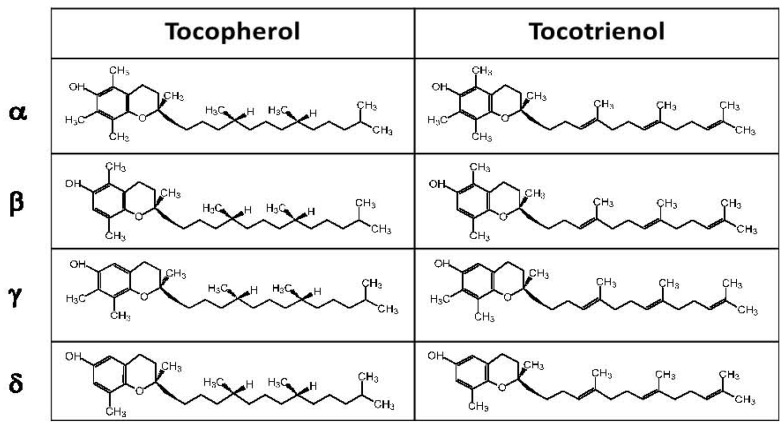
Tocopherols and tocotrienols. The isoforms differ in the number of methyl groups on the chromanol head group. Theα-tocopherol isoform is the most abundant in tissues because of preferential transfer of α-tocopherol in the liver by α-tocopherol transfer protein (α-TTP). The γ-tocopherol isoform is abundant in the diet but, in tissues, γ-tocopherol is 10 fold less abundant than α-tocopherol. The other forms of tocopherols and tocotrienols are less abundant in the diet and in tissues than α-tocopherol and γ-tocopherol.

The term vitamin E covers a group of eight lipid-soluble compounds: the α-, β-, γ-, and δ-tocopherols and the α-, β-, γ-, and δ-tocotrienols [11,12] (Figure 1). Mammals do not interconvert the tocopherol isoforms [6]. They are consumed in their original form from dietary plant lipids and loaded in intestinal-formed chylomicrons that are transported through the lymph to the liver [12,13]. *In vivo*, tocopherols are metabolized to carboxyethyl-hydroxychromans (CEHC) and are excreted [12,13]. The CEHC forms are reported to also have regulatory functions [14].

α-Tocopherol is the most biologically active form since it is specifically retained in the body by the liver α-tocopherol transfer protein (α-TTP). The α-TTP preferentially transfers α-tocopherol to lipid particles, resulting in 10 fold higher tissue levels of α-tocopherol compared to other tocopherols, reaching an average plasma concentration of 23 μM [15]. Due to lack of specific transfer mechanisms, other tocopherols and tocotrienols are not efficiently retained by the liver, and instead are metabolized, and predominantly eliminated [12] As a consequence, because the concentration of α-tocopherol is 10 fold higher in tissues than that of γ-tocopherol and the scavenging capacity of these tocopherols is relatively similar, total ROS scavenging capacity of α-tocopherol is also 10 fold higher than γ-tocopherol. Mice deficient in liver α-tocopherol transfer protein (αTTP) exhibit severe deficiency in tissue α-tocopherol as well as altered IgE and IL-5 after OVA challenge in the lung [16].

During lipid oxidation, the isoforms of vitamin E have similar capacity to scavenge ROS when present at equal molar concentrations [11,17,18]. This reaction produces oxidized tocopheroxyl radicals that can be recycled back to the active reduced form through reduction by vitamin C [19,20,21]. Without reduction of vitamin E by vitamin C, vitamin E can act as ROS donor [22]. In addition to scavenging ROS, γ-tocopherol, in contrast to α-tocopherol, also reacts with nitrogen species such as peroxynitrite, forming 5-nitro-γ-tocopherol [23,24,25]. Vitamin C is endogenously synthesized in mice whereas humans must obtain vitamin C directly from their diet [26]. Therefore, some clinical studies have supplemented patients with tocopherols and vitamin C [27,28,29,30].

Importantly, besides the antioxidant properties, it has been reported that tocopherols also have non-antioxidant signaling functions [5,11,31]. In agreement with this, we recently demonstrated that α-tocopherol and γ-tocopherol can bind protein kinase Cα (PKCα) and act as an agonist or antagonist, respectively, of PKCα activity [7]. Opposing functions of these tocopherols regulate recruitment of leukocytes during inflammation [4,5,9].

## 3. Experimental Evidence for Modulation of Lung Function by Vitamin E Isoforms

Asthma is characterized by inflammatory processes with T-helper (Th) cell responses of the Th2 phenotype being considered crucial for the initiation and perpetuation of the inflammatory responses [32]. Cytokines such as interleukin IL-4, IL-5, and IL-13 secreted by Th2 cells are important mediators of asthmatic and allergic inflammation that is characterized by elevated immunoglobulin E, mast cell degranulation, and eosinophilic inflammation [32]. During allergen challenge of lungs of guinea pigs, α-tocopherol and ascorbic acid levels are decreased in the bronchoalveolar lavage [33], suggesting that tocopherol supplementation may be beneficial in allergic disease.

Tocopherol supplementation during asthma or allergic inflammation has been evaluated in humans and animal models. Table 1 summarizes many of these studies. In animal studies of lung inflammation, there has been administration of various tocopherol isoforms in supplements and vehicles (Table 1A). Briefly, mice fed α-tocopherol starting two weeks before sensitization with OVA, a model allergen antigen, have a reduction in the number of eosinophils in the bronchoalveolar lavage (Table 1A line 2) [34]. In addition, Mabalirajan *et al*. [35] reported that oral administration of α-tocopherol after sensitization blocked OVA-induced lung inflammation (Table 1A line 3). In this report, α-tocopherol treatment reduced airway hyperresponsiveness, IL-4, IL-5, IL-13, OVA-specific IgE, eotaxin, TGFβ, 12/15-LOX, lipid peroxidation, and lung nitric oxide metabolites, while restoring cytochrome-c oxidase in lung mitochondria and bronchial epithelia [35]. Thus, in studies with supplementation with α-tocopherol without intake of γ-tocopherol, α-tocopherol alleviated allergic inflammation.

In our studies of tocopherol regulation of allergic inflammation, we used a model of allergic lung inflammation in which mice are sensitized and then challenged with the antigen chicken egg ovalbumin (OVA). We focused on supplementation with tocopherols after OVA antigen sensitization to determine whether tocopherol isoforms modulate the OVA antigen-challenge phase [5]. This design is clinically relevant because patients are already sensitized. Supplementation with tocopherols after OVA-sensitization and during OVA-challenge raised tissue tocopherols 5–7 fold higher compared to control mice without affecting body or lung weight [4,5]. The levels of tocopherols in our studies did not alter numbers of blood eosinophils, indicating that sufficient number of eosinophils was available for recruitment. Also, the expression of adhesion molecules, cytokines and chemokines required for the leukocyte recruitment was not compromised by tocopherol supplementation [5]. For leukocyte infiltration, we demonstrated opposing regulatory functions of α-tocopherol and γ-tocopherol during allergic lung inflammation in mice (Table 1A lines 1, 4–5) [4,5,6,36]. Specifically, the isoform α-tocopherol had anti-inflammatory properties and blocked airway hyperresponsiveness (Table 1A line 1), and the isoform γ-tocopherol was pro-inflammatory and increased airway hyperresponsiveness in mice (Table 1A line 4) [4,5,6]. Moreover, γ-tocopherol negated the anti-inflammatory benefit of α-tocopherol (Table 1A line 5) suggesting that these two tocopherols have competing opposing functions [5]. Physiological levels of d-γ-tocopherol, at only 10% the tissue concentration of d-α-tocopherol, ablates the anti-inflammatory benefit of the d-α-tocopherol isoform in response to OVA challenge [5]. This indicates that the level of γ-tocopherol in tissues is critical for α-tocopherol effectiveness at the time of supplementation after sensitization [5]. This modulation of leukocyte infiltration in allergic inflammation, without alteration of adhesion molecules, cytokines or chemokines, is similar to several previous reports of *in vivo* inhibition of lung inflammation by inhibition of intracellular signals in endothelial cells [37,38,39]. The competing functions of tocopherol isoforms have important implications for the interpretation of clinical and animal studies of vitamin E regulation of inflammation.

**Table 1:** α-Tocopherol (αT) and γ-tocopherol (γT) isoforms in lung studies.

**A. Animal**							
**Line**	**Airway Inflammation/Model**	**αT and γT Isoforms in the Studies**					**Major Outcome in Airway [reference]**
		**Tocopherol Isoform (dose)**		**Tocopherol Isoform (Figure 2 and [36]) in Reported Oil Vehicle**			
1	Eosinophil inflammation/mouse, OVA	αT (0.2 mg/20 g mouse/day × 8 days)		no tocopherolin ethoxylated castor oil			Beneficial, Eosinophil decrease [4,5]
2	Eosinophil inflammation/mouse, OVA	αT (500 mg/kg diet × 45 days)		tocopherol-stripped corn oil			Beneficial, Eosinophil decrease [34]
3	Eosinophil inflammation/mouse, OVA	αT (10 mg/kg mouse × twice/day × 14 days)		no tocopherol in ethanol			Beneficial, Eosinophil decrease [35]
4	Eosinophil inflammation/mouse, OVA	γT (0.2 mg/20 g mouse/day × 8 days)		no tocopherol in ethoxylated castor oil			Detrimental, Eosinophil increase [4,5]
5	Eosinophil inflammation/mouse, OVA	αT and γT (0.2 mg αT + 0.2 mg γT/20 g mouse/day × 8 days)		no tocopherol in ethoxylated castor oil			No effect [4,5]
6	Eosinophil inflammation/rat, OVA	αT (400 mg/kg/day × 10 days)		γT in soy oil			No effect [28]
7	Resolution of nasal eosinophilia/rat, OVA then tocopherol then Ozone	γT (100 mg/kg rat × 4 days)		tocopherol-stripped corn oil			Beneficial, Ozone-induced nasal inflammation [37]
8	Resolution of lung eosinophilia/rat, OVA then tocopherol & Ozone	γT (100 mg/kg rat × 4 days)		tocopherol-stripped corn oil			Beneficial, resolution of eosinophil inflammation [38]
9	Neutrophil inflammation/mouse, LPS	αT (50 mg/kg mouse × 1 day)					Beneficial, Neutrophil decrease [39]
10	Neutrophil inflammation/rat, LPS	αT (inhaled 30 µg/rat × 1 day)					Beneficial, Neutrophil decrease [40]
11	Neutrophil inflammation/rat, LPS	γT (30 mg/kg rat × 4 days)		tocopherol-stripped corn oil			Beneficial, Neutrophil decrease [41]
12	Neutrophil inflammation/rat, IL-1	αT (inhaled 30 µg/rat × 1 day)					Beneficial, Neutrophil decrease [42]
13	Neutrophil inflammation/rat, OVA	γT (100 mg/ kg rat × 2 days before OVA and 2 days after OVA)		tocopherol-stripped corn oil			Beneficial, Neutrophil decrease [43]
14	Neutrophil inflammation/sheep, burn & smoke	γT and αT (inhaled 1220 mg γT + 182 mg αT in 48 h)		γT in flaxseed oil			Beneficial, Neutrophil decrease [44]
**B. Human**							
**Line**	**Airway Clinical Condition**	**αT and γT Isoforms in the Studies**					**Major Outcome** **[reference]**
		**Tocopherol Isoform** **(Intake or Supplement Dose)**	**Isoforms (Figure 2) in** **Reported Oil Vehicle**	**Plasma Tocopherol [10,45,46,47]**			
				**Country**	**αT (μM)**	**γT (μM)**	
1	Asthma/lung function	αT intake (9.9 mg/day)		Italy	24	1.2	Beneficial [47,48]
2	Asthma/lung function	αT intake (6.7 mg/day)		Finland	24 or 41	0.5 or 1.8	Beneficial [48,49]
3	Asthma/lung function	αT intake (17.9 mg/day)		Netherlands	25	2.3	No effect [48]
4	Asthma	αT intake (3.3 to 17.1 or 209.8 mg/day)		USA	22 or 27	5 or 7	No effect [50]
5	Asthma	αT intake (1.1 to 15.7 mg/day)		UK	24 or 27	1.9 or 2.0	No effect [51]
6	Asthma/lung function	αT supplement (500 mg/day × 6 weeks)	γT in soy oil	UK	24 or 27	1.9 or 2.0	No effect [52]
7	Asthma	αT-acetate supplement (1000 mg/day × 16 weeks)		USA	22 or 27	5 or 7	Beneficial [53]
8	Asthma	αT supplement (500 mg/day) +Vitamin C supplement (2000 mg/day) × 12 weeks		USA	22 or 27	5 or 7	No effect [29]
9	Ozone/Asthma	Unknown isoforms in tocopherol supplement (50 mg/day) + Vitamin C (250 mg/day) × 12 weeks		Mexico	23 or 28	2.2 or 2.7	Beneficial [27]
10	Endotoxin (LPS)-induced neutrophil airway inflammation	isoform mixture in supplement (50 mg αT, 250 mg βT and δT, 540 mg γT)/day × 7 days	αT in sunflower oil	USA	22 or 27	5 or 7	Beneficial [41]

We have also demonstrated that tocopherol regulation of inflammation is partially reversible by supplemental levels of tocopherols but fully reversible by highly-elevated levels (10× supplemental levels) of tocopherols. However, the implications and adverse effects of higher than supplemental levels of tocopherol should be carefully taken into account. Some reports indicate that high doses of tocopherol (≥400 IU/day) can significantly increase hemorrhagic stroke, elevate blood pressure, and increase all-cause mortality [40,41,42,43,44,45]. Consequently, administration of high-dose α-tocopherol may be a potentially risky approach for reversing the pro-inflammatory effects of supplemental levels of γ-tocopherol. More recently, a meta-analysis that reexamines the relationship between supplemental vitamin E and all-cause mortality showed supplementation with vitamin E appears to have no effect on all-cause mortality at doses up to 5500 IU/day [46]. In summary, at physiological tissue concentrations, these natural tocopherol isoforms have distinct regulatory effects on leukocyte recruitment in allergic inflammation [4].

**Figure 2:**
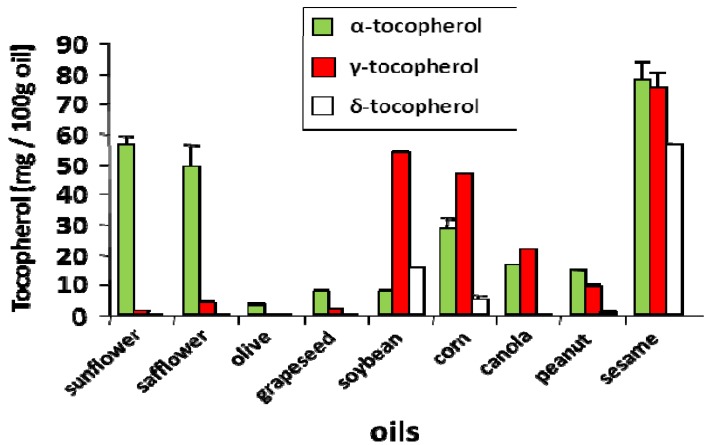
α-Tocopherol and γ-tocopherol in dietary oils. Adapted from [5]. Tocopherols from dietary oils (sunflower oil from Spectrum Organic Products, LLC; safflower oil from Spectrum; olive oil from Colavita; soybean oil from Crisco; corn oil from Mazola; grapeseed oil from Kusha, Inc.; peanut oil from Essentials by Supervalu; canola oil from Crisco; sesame oil from Lavita). Tocopherols were extracted from dietary oils and measured by HPLC with an electrochemical detector as previously described [5].

Discrepancy among the reports for tocopherol regulation of lung inflammation in animals may be also explained by another important parameter such as the oil vehicle used during supplementation. We and others have determined the levels of α-tocopherol and γ-tocopherol in dietary oils by HPLC (Figure 2) [5,13,47]. High content of γ-tocopherol is found in soybean, corn, canola and sesame oils, therefore administration of supplemental tocopherols in any of these vehicles may lead to misinterpretations. For example, Suchankova *et al*. reported that the administration of purified α-tocopherol in soy oil by gavage had no major effect on immune parameters or lung airway responsiveness in mice challenged with OVA (Table 1A line 6) [28]. In this study, our interpretation is that high γ-tocopherol in the soy oil vehicle negated the effect of the α-tocopherol, although tissue and plasma tocopherol levels were not measured.

It is reported that γ-tocopherol is beneficial for resolution of ozone-induced airway inflammation or acute endotoxin-induced neutrophil airway inflammation (Table 1A line 7–11). Endotoxin and ozone induce increases in reactive nitrogen species [48,49], which are scavenged by γ-tocopherol but not α-tocopherol [23,24,25]. In a study examining γ-tocopherol modulation of ozone exposure after OVA challenge, there was reduced lung and nasal eosinophils in rats receiving OVA and then γ-tocopherol for 4 days and then challenged with ozone (Table 1A line 8) [50,51]. However, γ-tocopherol was administered after OVA challenge and it takes a few days to raise tissue tocopherol levels, which in this protocol is after the peak of eosinophil infiltration. Therefore, the effect on eosinophils at 4 days after the last OVA challenge was during the resolution phase of eosinophil inflammation (Table 1A line 8). In asthmatic children exposed to ozone, vitamin E and C supplementation reduces IL-6 in nasal lavages, although the isoform of vitamin E was not indicated (Table 1B line 9) [27]. In another report, inflammation that was primarily neutrophils was inhibited in rats receiving one OVA sensitization then γ-tocopherol in tocopherol-stripped corn oil for four days starting two days before two consecutive days of OVA challenges (Table 1A line 13) [52]. In a LPS-induced or IL-1-induced lung inflammation model, administration of aerosol α-tocopherol (30 µg/rat) immediately before inhalation of LPS or IL-1 reduces lung neutrophilia, TNF-α and cytokine-induced neutrophil chemoattractant-1 (CINC-1) (Table 1A line 10, 12) [53,54]. However, the source and purity of the α-tocopherol, as well as lung tissue levels of tocopherols, were not determined. Lung neutrophilia was also reduced in an LPS inflammation model when liposomes containing α-tocopherol (50 mg α-tocopherol/kg mouse) were administered; however, there was no effect on blood neutrophil numbers, TNFα, IL-1β or MIP-1α (Table 1A line 9) [55]. Furthermore, nebulized γ-tocopherol and α-tocopherol in flaxseed oil containing tocopherols [56] reduced neutrophilia, IL-8 and IL-6 in burn and smoke inhalation injury model in sheep (Table 1A line 14) [57]. In humans, short-term administration of a mixture of γ-tocopherol and α-tocopherol was beneficial for acute LPS-induced neutrophil inflammation (Table 1B line 10) [58]. In summary, differences among the reports of tocopherol regulation of lung inflammation likely reflect differences in the type of inflammation, isoforms of tocopherols and vehicle used, tocopherol isoform concentrations, basal level of tocopherol in tissues and time of administration of tocopherols.

Furthermore, at the molecular level, we have demonstrated an *in vitro* mechanism for the opposing functions for α-tocopherol and γ-tocopherol on leukocyte recruitment in the mouse lung. During allergic inflammation, leukocytes are recruited from the blood into the tissues by migrating across the vascular endothelial cells. We have demonstrated that the migration of leukocytes across endothelial cells is inhibited by pretreatment of the endothelial cells with α-tocopherol and elevated by pretreatment of the endothelial cells with γ-tocopherol [5]. Endothelial cells pretreated with α-tocopherol plus γ-tocopherol resulted in an intermediate phenotype [5]. The opposing functions of α-tocopherol and γ-tocopherol on endothelial cells during leukocyte transendothelial migration can occur through direct regulation of mediators of signal transduction. Briefly, the recruitment of the leukocytes to sites of allergic inflammation requires leukocyte binding to endothelial cell adhesion molecules such as VCAM-1 and ICAM-1. These adhesion molecules signal through protein kinase Cα (PKCα) for the recruitment of leukocytes [36,59]. We have previously demonstrated that α-tocopherol inhibits VCAM-1 and ICAM-1 activation of PKCα in endothelial cells and this inhibition is opposed by pretreatment of endothelial cells with γ-tocopherol [36,59]. α-Tocopherol has been also reported to inhibit PKCα activation in other cell systems or cell extracts, but the mechanisms for inhibition are not known [60]. We have demonstrated that, in endothelial cells, VCAM-1 dependent leukocyte migration requires PKCα activation by oxidation, and pretreatment of endothelial cells with physiological concentration of α-tocopherol but not γ-tocopherol inhibits PKCα oxidation [59] and leukocyte migration [5]. Moreover, pretreatment of human lung microvascular cells with α-tocopherol inhibits PKCα activation through xanthine oxidase and ERK1/2 [36]. Also, we demonstrated that recombinant PKCα is directly regulated by tocopherols [7]. α-tocopherol and γ-tocopherol both bind the C1a regulatory domain of PKCα. Upon binding to PKCα, α-tocopherol decreases and γ-tocopherol increases recombinant PKCα activity [7]. Thus, α-tocopherol functions as an antagonist and γ-tocopherol functions as an agonist of PKCα [7]. In summary, PKCα is differentially regulated by tocopherol isoforms in endothelial cells, which is critical for leukocyte recruitment in allergic lung inflammation and airway hyperresponsiveness.

## 4. Tocopherol Isoforms and Their Clinical Relevance

Patients with asthma have reduced α-tocopherol and ascorbic acid in airway fluid but the average plasma concentration of α-tocopherol and ascorbic acid in these patients is normal [61,62]. Interestingly, countries with the highest prevalence rate of asthma tend to have higher plasma levels of γ-tocopherol [10,63,64,65]. In the Unites States, the average human plasma γ-tocopherol levels are 2 to 5 times higher than those of many European and Asian countries, whereas the average levels of human plasma α-tocopherol is similar among all countries [10,47]. Most vitamin E in the diet in the United States comes in the form of γ-tocopherol from the major dietary vegetable oil in the US, soybean oil [66]. Therefore, the high human plasma γ-tocopherol levels in the US population are consistent with high levels of γ-tocopherol in this oil (Figure 2) [10,13,67,68]. Soybean oil administration in humans and hamsters increases the plasma γ-tocopherol levels 2–5 fold [69,70]. In our murine models of allergic asthma, we achieved this fold increase in plasma γ-tocopherol levels by dietary supplementation, which in turn resulted in exacerbation of lung eosinophil inflammation and suppression of the anti-inflammatory functions of α-tocopherol [5].

γ-Tocopherol is low in other oils such as sunflower and olive oil, commonly used across Europe (Figure 2) [5]. This is reflected in clinical studies where α-tocopherol supplementation of asthmatic patients in Italy and Finland was beneficial for outcomes of incidence of physician diagnosed asthma, and lung function as measured by forced expiratory volume in one second (FEV1) or wheeze (Table 1B lines 1, 2) [71,72,73,74,75]. Disappointingly, α-tocopherol was not beneficial for asthmatic patients in the United States or the Netherlands [71,72,73,74,75], which have high plasma levels of γ-tocopherol (Table 1B lines 3, 4). A meta-analysis study indicated that vitamin E had no association with asthma outcome measures of lung function and wheeze [76] but this did not take into account the opposing functions of α-tocopherol and γ-tocopherol. In contrast, another study in the United States reported that administration of a very high dose of α-tocopherol acetate (1500 IU which is 1006 mg) to mild atopic asthmatics for 16 weeks increased plasma α-tocopherol, decreased plasma γ-tocopherol, and improved airway responsiveness to methacholine challenge (Table 1B line 7) [77].

As countries assume Western lifestyles, diets may change, including increased consumption of soybean oil containing γ-tocopherol (Figure 2) [66]. Two Scottish birth cohorts have now reported that reduced maternal intake of vitamin E (likely referring to α-tocopherol) was associated with increased asthma and wheezing in children up to 5 years old [78,79]. Coincidentally, the same report mentioned that, in the last 37 years, there was a significant increase in vegetable oil intake by Scottish population [78]. The therapeutic potential of dietary manipulation and supplementation in children with asthma requires further work. The investigation of early life diet in relation to childhood asthma raises the possibility of early life dietary intervention to prevent asthma. In addition, in a study in the United Kingdom, α-tocopherol administration in soybean oil to asthmatics did not have benefit (Table 1B line 6) [80]. These differences in outcome of the clinical reports mirror the opposing regulatory functions of α- and γ-tocopherol forms of vitamin E consumed in diets, supplements and supplement vehicles [5]. Our interpretation, supported by animal studies, is that the γ-tocopherol in soybean oil or other vehicles ablates the benefit of α-tocopherol supplementation [5]. Of interest, in a recent study, *ex vivo* treatment of human macrophages with high levels (300 µM) of γ-tocopherol decreased macrophage phagocytosis via modulation of surface receptor activity [81]. However, such highly-elevated γ-tocopherol levels are not achievable *in vivo*. Importantly, although high levels of tocopherols have been used clinically, they can have adverse effects on the brain in animals and humans [81]. Moreover, most clinical studies on vitamin E include mixed forms of natural or synthetic tocopherols for dietary supplementation (Table 1B lines 8–10), which complicates outcomes even further due to unknown interaction effects of multiple isoforms at different concentrations. It is suggested that, since levels of α-tocopherol and other antioxidants are low in asthmatics [61,62,82,83,84] and α-tocopherol can reduce inflammation, supplementation with physiological levels of natural α-tocopherol and maintenance of low dietary levels of γ-tocopherol may be an attractive strategy to promote an optimal anti-inflammatory balance in asthmatics. Nevertheless, outcomes from clinical trials reflect the opposing regulatory functions of α- and γ-tocopherol forms of vitamin E consumed in diets, supplements and supplement vehicles (Table 1B) [5].

In addition to opposing regulatory functions of tocopherol isoforms in allergic disease, there are also conflicting outcomes for vitamin E in other inflammatory diseases, including arthritis and cardiovascular disease. Briefly, human plasma γ-tocopherol positively associates with osteoarthritis, whereas plasma α-tocopherol negatively associates with osteoarthritis [85]. In coronary heart disease and stroke, studies of tocopherols and heart disease are complex, because different dietary oils contain different lipids that affect heart disease. Nevertheless, for plasma γ-tocopherol, it is either not associated with heart disease or is associated with an increase in risk for myocardial infarction [86]. In contrast, for α-tocopherol, it is either not associated with heart disease or is associated with reduced death from heart disease [87,88,89,90]. Therefore, for those reports with an effect on heart disease, γ-tocopherol associates with an increase and α-tocopherol associates with a decrease in heart disease. In summary, opposing functions of α-tocopherol and γ-tocopherol are consistent with the outcomes for the clinical studies of tocopherol isoforms in heart disease and asthma.

## 5. Conclusions

Epidemiological studies and randomized prevention trials have demonstrated the potential of a number of protective dietary factors for asthma, including vitamin E. However, these reports indicate seemingly contradictory outcomes for supplementation with the α-tocopherol and γ-tocopherol isoforms of vitamin E. These discrepancies in clinical results are consistent with experimental evidence of differential regulatory activity of these tocopherol isoforms in animal asthma models. We and others demonstrated that tocopherols function beyond their antioxidant capacity and regulate signaling pathways essential in the inflammatory process. Specifically, supplementation with physiological levels of purified natural forms of the vitamin E isoforms α-tocopherol and γ-tocopherol has opposing regulatory functions during allergic inflammation such that α-tocopherol is anti-inflammatory and γ-tocopherol is pro-inflammatory. In conclusion, understanding of the differential regulation of inflammation by isoforms of vitamin E provides a basis towards designing drugs and diets that more effectively modulate inflammatory pathways and improve lung function in disease.

## Abbreviations

CEHC: carboxyethyl-hydroxychroman
FEV1: forced expiratory volume in one second
ICAM-1: intercellular adhesion molecule-1
OVA: chicken egg ovalbumin
PKCα: protein kinase Cα
ROS: reactive oxygen species
VCAM-1: vascular cell adhesion molecule-1
αTTP: α-tocopherol transfer protein
VCAM-1: vascular cell adhesion molecule-1
